# CD36 haplotypes are associated with lipid profile in normal-weight subjects

**DOI:** 10.1186/1476-511X-12-167

**Published:** 2013-11-05

**Authors:** Luz E Ramos-Arellano, Aralia B Salgado-Bernabé, Iris P Guzmán-Guzmán, Lorenzo Salgado-Goytia, José F Muñoz-Valle, Isela Parra-Rojas

**Affiliations:** 1 Laboratorio de Investigación en Obesidad y Diabetes, Unidad Académica de Ciencias Químico Biológicas, Universidad Autónoma de Guerrero, Chilpancingo, Guerrero, México; 2 Departamento de Biología Molecular y Genómica, Instituto de Investigación en Ciencias Biomédicas, Centro Universitario de Ciencias de la Salud, Universidad de Guadalajara, Jalisco, México

**Keywords:** Dyslipidemia, *CD36* gene, Polymorphisms, Haplotypes

## Abstract

**Background:**

Dyslipidemia is a common metabolic disorder that may result from abnormalities in the synthesis, processing and catabolism of lipoprotein particles. Disorders of lipoprotein concentrations and elevated concentration of oxidized lipoproteins (oxLDL) are risk factors in the pathogenesis of cardiovascular diseases (CVD). CD36 plays an important role in lipid metabolism and polymorphisms in the *CD36* gene are related to cardiovascular risk factors. The purpose of this study was to evaluate whether there is an association between genotypes and haplotypes of five polymorphisms in the *CD36* gene with lipid levels in young normal-weight subjects.

**Methods:**

A total of 232 unrelated subjects with normal-weight of 18 to 25 years old (157 women and 75 men) were randomly selected. The lipid profile and glucose levels were measured by enzymatic colorimetric assays. Genotyping of the polymorphisms -33137A/G (rs1984112), -31118G/A (rs1761667), -22674 T/C (rs2151916), 27645 Ins/Del (rs3840546) and 30294G/C (rs1049673) in the *CD36* receptor gene was performed by polymerase chain reaction and restriction fragment length polymorphism, linkage disequilibrium analysis among the five polymorphisms and an analysis of haplotype were estimated.

**Results:**

HDL-C levels was lower in men than in women (*P* = 0.03). However, the median oxLDL levels in men was higher than in women (*P* = 0.05). There was no significant difference in the levels of TC, TG, LDL-C and glucose (*P* > 0.05). HDL-C levels were lower in the subjects with TC genotype of polymorphism -22674 T/C (*P* = 0.04), but the carriers of TT genotype had lower oxLDL levels (*P* = 0.01). LDL-C levels were higher in young carriers of CC genotype for 30294G/C polymorphism than non-carriers (*P* = 0.03). The subjects carrying the AATDC haplotype had 3.2 times presumably higher risk of LDL-C > 100 mg/dL than the carrying the AGTIG haplotype (*P* = 0.02), whereas the subjects carrying the AATIC haplotype had 2.0 times presumably higher risk of TC > 200 mg/dL than the carrying the AGTIC haplotype (*P* = 0.02).

**Conclusion:**

The study provides evidence of a genetic association of *CD36* haplotypes with the variability in LDL-C and TC levels in a sample of normal-weight subjects.

## Background

Dyslipidemia is a common metabolic disorder that may result from abnormalities in the synthesis, processing and catabolism of lipoprotein particles [1]. Lipoproteins are the macromolecular vehicles for transport of hydrophobic lipids throughout the aqueous environment of the circulatory system; they are composed of various lipid species aggregated with specific proteins (apolipoproteins), which act as receptor ligands, stabilize the emulsion and confer structural properties to the lipoprotein particle [2].

Lipid transport and metabolism involves three general pathways: (1) the exogenous pathway, whereby chylomicrons are synthesized by the small intestine, and dietary triglycerides (TGs) and cholesterol are transported to various cells of the body; (2) the endogenous pathway, whereby the very low-density lipoprotein cholesterol (VLDL-C) and TGs are synthesized by the liver for transport to various tissues; in the plasma, the TGs in VLDLs are hydrolyzed by lipoprotein lipase (LPL), generating a series of smaller, cholesterol enriched lipoproteins: intermediate-density lipoprotein (IDL) and low-density lipoprotein (LDL); (3) and the reverse cholesterol transport, whereby high-density lipoprotein (HDL) in a series of metabolic steps facilitates the removal of cholesterol from the peripheral tissues for delivery to the liver and steroidogenic organs [3]. Disorders of lipoprotein concentrations such as elevated LDL-C (≥160 mg/dL), low HDL-C (<40 mg/dL), increase TG (≥150 mg/dL) and elevated oxidized lipoproteins (oxLDL) levels are risk factors in the pathogenesis of cardiovascular diseases (CVD) [4]. However, it has been shown that atherosclerosis can begin with LDL-C levels ≥100 mg/dL, therefore modification of lipid and lipoprotein classification identifies to LDL-C <100 mg/dL as optimal [5].

CD36 is a polypeptide from 78 to 88 kDa of molecular weight (50 kDa deglycosylated) depending on the cell type [6]. CD36 receptor is involved in a variety of biological processes including lipid metabolism, inflammation, atherosclerosis, angiogenesis, innate immune responses, uptake of apoptotic cells, oxidized lipids and advanced glycation end products, transforming growth factor-β activation, insulin resistance, diabetes and thrombosis, depending on nature of the ligand and tissue or cell type on which it is expressed [7-10]. CD36 contributes to oral fat perception and intestinal chylomicron formation [11]. CD36 is a multi-ligand scavenger receptor expressed on a variety of cell types including adipocytes, myocytes, monocytes, macrophages, platelets, hepatocytes and vascular epithelial cells [12,13]. Given the many functions of CD36 including long chain fatty acid transport (LCFA), changes in CD36 expression and protein may lead to several disturbances including insulin resistance and dyslipidemia [14]. This receptor is up regulated by oxLDL in macrophages and contributes to the formation and accumulation of foam cells at sites of arterial lesions during early and late atherosclerosis [15].

The *CD36* gene is located on chromosome 7 q11.2, is encoded by 15 exons and spans 36 Kb, CD36 plays an important role in lipid metabolism and its gene polymorphisms are associated to cardiovascular risk factors [16,17]. Genome-wide linkage scans have identified nearby regions of chromosome 7 that are associated with features of metabolic syndrome, such as triglyceride concentrations, HDL-C, and triglyceride/HDL ratio [18].

The aim of this study was to evaluate whether there is an association between genotypes and haplotypes of five polymorphisms in the *CD36* gene with lipid levels in normal-weight young men and women.

## Results

### General and biochemical characteristics

General and biochemical characteristics of study subjects according to gender are shown in Table 1. The measurements of body weight, height, systolic blood pressure, prevalence of hypertension and oxLDL levels were higher in men than in women (*P* < 0.05), whereas HDL-C levels was lower in men than in women (*P* = 0.03). There was no significant difference in the levels of TC, TG, LDL-C and glucose (*P* > 0.05).

**Table 1:** Clinical characteristics and lipid levels by gender

**Variables**	**Female (n = 157)**	**Male (n = 75)**	***P *** **value**
Age (years)	21 (19–22)	20 (20–22)	0.433
Weight (kg)	52 (48–56)	63 (57–68)	0.001
Height (cm)	156 (152–159)	169 (166–173)	0.001
BMI (kg/m^2^)	22 (20–23)	22 (20–23)	0.411
SBP (mmHg)	102 (97–106)	110 (103–119)	0.001
DBP (mmHg)	66 (60–71)	68 (61–72)	0.299
Hypertension (%)	4 (3)	8 (11)	0.009
TC (mg/dL)	75 (58–102)	77 (57–19)	0.926
HDL-C (mg/dL)	48 (38–58)	42 (37–52)	0.032
LDL-C (mg/dL)	93 (67–118)	89 (70–116)	0.311
oxLDL (U/L)	34 (27–46)	39 (31–53)	0.053
TG (mg/dL)	75 (58–103)	77 (57–109)	0.926
Glucose (mg/dL)	81 (75–88)	81 (75–88)	0.942

*SBP*, Systolic blood pressure; *DBP*, Diastolic blood pressure; *TC*, Total cholesterol; *HDL-C*, High-density lipoprotein cholesterol; *LDL-C*, Low-density lipoprotein cholesterol; *oxLDL*, Oxidized low-density lipoprotein; *TG*, Triglyceride. The values were presented as median (percentile 25-75^th^). The difference between genders was determined by the Wilcoxon-Mann–Whitney test. Data of hypertension were presented in (n) and the percentages; the difference between genders was determined by chi-square test.

### Genotypic and allelic frequencies

**Figure 1:**
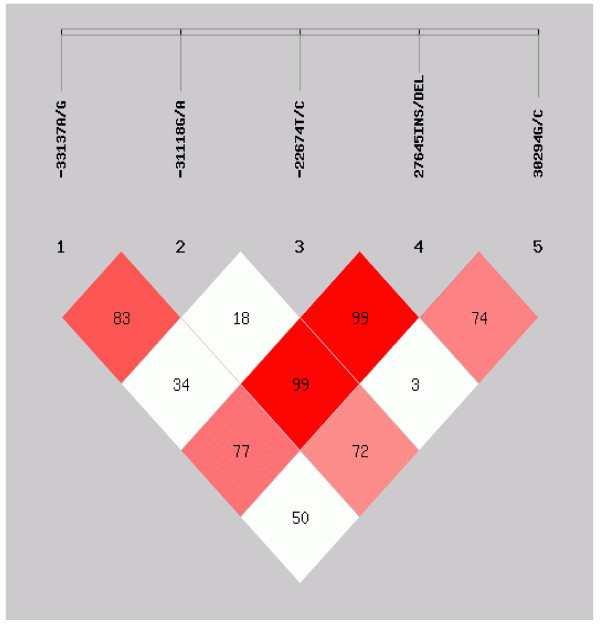
**Linkage disequilibrium test of *CD36 *** **gene polymorphisms (D’).**

Table 2 shows the genotype and allelic distribution of five *CD36* polymorphisms in normal-weight subjects, genotype frequencies of each polymorphism were in Hardy-Weinberg equilibrium. The 27645Ins/Del polymorphism was found high linkage disequilibrium (LD) with -31118G/A and -22674 T/C polymorphisms; while the -33137A/G and -31118G/A polymorphisms were also found in high LD (Figure 1).

**Table 2:** Allele and genotype frequencies in normal-weight subjects

**Polymorphism**	**Genotype n (%)**	**Allele**	**n (%)**	**HWE *X***^***2 ***^ ***(P value)***	**Location**
-33137A/G					5′flanking exon 1A
AA	117 (50)	A	328 (0.71)	0.11 (0.73)	
AG	94 (41)	G	136 (0.29)		
GG	21 (9)				
-31118G/A					5′flanking exon 1A
GG	46 (20)	G	196 (0.42)	1.53 (0.21)	
GA	104 (45)	A	268 (0.58)		
AA	82 (35)				
-22674 T/C					Promoter
TT	106 (46)	T	309 (0.67)	0.84 (0.35)	
TC	97 (42)	C	155 (0.33)		
CC	29 (12)				
27645Ins/Del					Exon 14 (3′-UTR)
Ins/Ins	206 (89)	Ins	437 (0.94)	0.06 (0.79)	
Ins/Del	25 (10.6)	Del	27 (0.06)		
Del/Del	1 (0.4)				
30294G/C					Exon 15 (3′-UTR)
GG	13 (6)	G	111 (0.24)	0.01 (0.92)	
GC	85 (36)	C	353 (0.76)		
CC	134 (58)				

*HWE*, Hardy-Weinberg Equilibrium.

As shown in Table 3, the levels of HDL-C were lower in the -22674 TC carriers (*P* = 0.04), but the subjects with TT genotype for this same polymorphism had lower oxLDL levels than non-carriers (*P* = 0.01). LDL-C levels were higher in 30294 CC carriers than non-carriers (*P* = 0.03).

**Table 3:** **Blood lipid profile according to *CD36 *** **polymorphisms**

**Polymorphism**	**TC (mg/dL)**	**TG (mg/dL)**	**HDL-C (mg/dL)**	**LDL-C (mg/dL)**	**oxLDL (U/L)**
-33137A/G					
AA	150 (140–178)	70 (53–93)	43 (34–52)	86 (59–124)	32 (26–39)
AG	157 (138–180)	79 (56–109)	41 (35–49)	73 (48–109)	30 (24–38)
GG	154 (136–176)	66 (52–109)	41 (35–50)	63 (52–89)	33 (26–35)
*P* value	*0.64*	*0.40*	*0.88*	*0.19*	*0.70*
-31118G/A					
GG	149 (131–171)	70 (53–102)	44 (34–59)	82 (55–103)	30 (26–35)
GA	160 (144–181)	79 (63–109)	41 (36–48)	75 (51–109)	31 (26–45)
AA	152 (140–172)	63 (53–95)	42 (34–51)	87 (57–137)	30 (25–38)
*P* value	*0.18*	*0.09*	*0.71*	*0.20*	*0.34*
-22674 T/C					
TT	152 (137–169)	65 (52–109)	43 (37–58)	77 (51–114)	28 (25–35)
TC	154 (138–179)	77 (62–102)	38 (32–48)	89 (61–116)	34 (28–43)
CC	153 (145–175)	71 (53–92)	44 (34–52)	73 (52–106)	34 (28–46)
*P* value	*0.66*	*0.71*	*0.04*	*0.60*	*0.01*
27645Ins/Del					
Ins/Ins	153 (139–179)	72 (53–103)	42 (34–50)	78 (55–105)	31 (26–38)
Ins/Del	152 (136–169)	68 (56–108)	42 (31–50)	111 (54–145)	29 (26–34)
Del/Del	149 (149–149)	129 (129–129)	34 (34–34)	123 (123–123)	-
*P* value	*0.66*	*0.43*	*0.60*	*0.31*	*0.51*
30294G/C					
GG	134 (114–150)	77 (63–107)	46 (39–59)	72 (56–103)	33 (26–47)
GC	153 (133–179)	76 (58–106)	46 (36–57)	89 (67–112)	35 (28–46)
CC	150 (130–175)	75 (56–103)	46 (38–56)	96 (75–121)	36 (28–48)
*P* value	0.06	0.89	0.76	0.03	0.72

*TC*, Total cholesterol; *TG*, Triglyceride; *HDL-C*, High-density lipoprotein cholesterol; *LDL-C*, Low-density lipoprotein cholesterol; *oxLDL*, Oxidized low-density lipoprotein. The values were presented as median (percentile 5-95^th^). *P* values among genotypes by Kruskal Wallis test.

### Haplotypes and dyslipidemia susceptibility

To examine the combined effect of five variants -33137A/G (rs1984112), -31118G/A (rs1761667), -22674 T/C (rs2151916), 27645Ins/Del (rs3840546) and 30294G/C (rs1049673) in the *CD36* receptor gene locus, we performed haplotype analysis considered the combinations most frequent (Table 4). In particular, the subjects with AATDC (12122) haplotype had 3.2 times higher risk of LDL-C >100 mg/dL than the carrying the AGTIG (11111) haplotype (*P* = 0.02), whereas the subjects carrying the AATIC (12112) haplotype had 2.0 times presumably higher risk of TC > 200 mg/dL than the carrying the AGTIC (11112) haplotype (*P* = 0.02).

**Table 4:** **Association of *CD36 *** **haplotypes with lipid levels**

**Haplotype**	**Cases (n = 96) LDL-C >100 mg/dL**	**Controls (n = 134)**	**OR (95% CI)**	***P *** **value**
AGTIG	(0.046)	(0.035)	1.28 (0.50-3.28)	0.59
11111				
AGTIC	(0.088)	(0.081)	1.06 (0.54-2.08)	0.84
11112				
AATIC	(0.365)	(0.340)	1.08 (0.73-1.61)	0.68
12112				
AATDC	(0.060)	(0.019)	3.25 (1.12-9.40)	0.02
12122				
AACID	(0.116)	(0.145)	0.75 (0.42-1.31)	0.31
12212				
GGTIG	(0.045)	(0.099)	0.42 (0.19-0.92)	0.02
21111				
GGTIC	(0.042)	(0.034)	1.21 (0.46-3.20)	0.69
21112				
GGCIG	(0.050)	(0.086)	0.55 (0.25-1.19)	0.12
21211				
GGCIC	(0.112)	(0.066)	1.74 (0.90-3.37)	0.09
21212				
**Haplotype**	**Cases (n = 26) Cholesterol total >200 mg/dL**	**Controls (n = 206)**	**OR (95% CI)**	***P *** **value**
AGTIC	(0.052)	(0.087)	0.60 (0.16-2.15)	0.43
11112				
AGCIC	(0.031)	(0.012)	2.86 (0.46-17.50)	0.23
11212				
AATIC	(0.472)	(0.332)	2.02 (1.09-3.74)	0.02
12112				
AATDC	(0.020)	(0.044)	0.46 (0.06-3.43)	0.44
12122				
AACIC	(0.109)	(0.131)	0.85 (0.33-2.13)	0.72
12212				
GGTIG	(0.021)	(0.081)	0.25 (0.037-1.74)	0.13
21111				
GGTIC	(0.022)	(0.039)	0.58 (0.086-3.95)	0.57
21112				
GGCIG	(0.083)	(0.068)	1.30 (0.45-3.77)	0.62
21211				
GGCIC	(0.080)	(0.088)	0.94 (0.32-2.74)	0.92
21212				

1 = Major allele, 2 = minor allele. Markers: -33137A/G, -31118G/A, -22674 T/C, 27645Ins/Del, 30294G/C polymorphisms of *CD36* gene.

## Discussion

The results of the present study show that the levels of serum HDL-C in a Mexican young population with normal-weight were higher in women than in men. This gender difference in HDL-C may be attributed to high endogenous estrogen levels in women, as it has been demonstrated that estrogen concentrations may increase HDL-C, which confers a protective effect of cardiovascular disease to premenopausal women [19]. However, also has been shown that the difference in HDL-C between women and men is an androgen effect, but not an estrogen effect, therefore, at puberty, concurrent with the rise in endogenous testosterone levels, the HDL-C levels in young men decline to the adulthood [20].

There was no significant difference in the levels of TC, TG, and LDL-C between genders. These findings are in agreement with previous studies in other populations [21-23]. In this research, oxLDL levels were higher in men than women; but there is a lack of published data about the oxLDL levels by gender. However, it has been shown that men have a higher oxidative stress compared to women, due to an increased generation of reactive oxygen species (ROS) and/or reduced activity of antioxidants, considering that under healthy conditions, cellular respiration in the mitochondria is the dominant source of ROS, therefore, a higher baseline metabolic rate in men than in women might contribute to a higher level of oxidative stress in men [24]. It is also known that an increase in oxLDL, as a consequence of increased oxidative stress and reduced antioxidant defenses, is a key event in the atherogenic process and a cardiovascular disease risk factor, which may be reflected in an increase in circulating levels of oxLDL [25].

*CD36* gene is highly polymorphic; data from Ensembl Variation Build 60 (which is based on dbSNP Release 131) describe 2935 common genetic variants within 5 kb of the gene. Some involve putative transcription factor binding sites or sites in the 5′-untranslated region, which are of potential significance because translational efficiency of the CD36 mRNA and thereby CD36 protein expression levels have been shown to be regulated by variants in the 5′-untranslated region [26]. A study on *CD36* gene variants showed that subjects with GG homozygote genotype of the variant 80121259A > G (rs3211849) had a higher triglyceride level (99.16 ± 2.61 mg/dL) compared with non-carriers (89.27 ± 1.45 mg/dL, *P* = 0.001). In addition, compared with non-carriers, subjects with CT heterozygous and TT homozygous genotypes of the variant 80122878C > T (rs1054516) had a significantly lower HDL-C level (46.6 ± 0.46 mg/dL for non-carriers, 44.6 ± 0.36 mg/dL for heterozygous, and 44.3 ± 0.56 mg/dL for homozygous, *P* = 0.0008) [27].

Previous genetic studies showed various effects between *CD36* locus and dyslipidemias, a genome-wide linkage scan among 418 individuals from 27 extended Mexican American families found two different locations on chromosome 7 were suggested as linked to susceptibility loci influencing in HDL cholesterol and triglycerides levels, however, it has been reported a major susceptibility locus in chromosome 15q influencing in TG levels in a Mexican American population [28]. In addition, among non-diabetic Mexican American families, quantitative trait locus study showed a strong linkage of two factors metabolic syndrome related, HDL-C and triglycerides to chromosome 7 (LOD score up to 3.2) [29].

A meta-analysis showed that *CD36* gene locus (7p11-q21.11) was significantly linked to triglycerides and triglycerides/HDL-C ratio, but not linked to LDL or total cholesterol [30]. In addition, a study in 61 *CD36*-deficient patients and 25 controls showed that the HDL-C concentrations in the *CD36*-deficient patients were significantly higher than in the control subjects, however, nondiabetic *CD36*-deficient patients had higher triglyceride concentrations than the control subjects, and triglyceride concentrations were higher in the diabetic CD36-deficient patients than in the nondiabetic *CD36*-deficient patients [31].

In this research the genotype frequencies of each polymorphism were in Hardy-Weinberg equilibrium. However, genotype and allele frequencies found in this study for each of the polymorphisms in the *CD36* gene are different to those reported in Caucasian population [32,33]. Mexicans are an admixed population, descended from a recent mix of Amerindian and European ancestry with a small proportion of African ancestry [34]. The influence of various races in our genetic background as Mexican population may explain the allele and genotype differences with respect to other populations.

In this study, the 27645Ins/Del polymorphism was found in high LD with polymorphisms -31118G/A, -22674 T/C and 30294G/C. These results were similar to those previously reported by Ma X, *et al.* in Caucasians [32].

Here we show an association between the *CD36* polymorphisms and serum lipid levels in Mexican subjects. In this study, the subjects with -22674TC genotype had lower HDL-C levels than non-carriers. However, oxLDL levels were lower in the TT subjects; these results are different to those reported by Goyenechea *et al.*, 2008 in the Spanish population, they shown that subjects carrying the CC genotype had higher levels of HDL-C and lower LDL-C, this difference may be due to they did a study with dietary intervention where participants were under a low calorie diet for 8 weeks and the sample size, also their sample size was larger than ours, they concluded that the association with lower LDL-C level observed in response to the low calorie diet may devolve from reduced ability of –22674CC homozygotes to take up fatty acids from the intestine, to synthesize triglycerides and secrete LDL-C. Our study also found that 30294CC carriers had higher LDL-C concentrations compared with subjects with the other genotypes; CC genotype was associated with high levels of free fatty acids in Caucasian population [9].

Although it is not clear how -33137A/G, -31118G/A -22674 T/C, 27645Ins/Del and 30294G/C polymorphisms can modulate lipid metabolism, has been demonstrated that -22674 T/C SNP is located upstream of the promoter, 14 bases 5′of the transcription start site, which is a binding element for the transcriptional repressor GFI1B, this SNP was in complete LD with the -33137A/G SNP in Caucasians from the general population, thus so has been reported that the allele A at position -33137A/G is in complete LD with the in3(TG)_13_ variant, which determines the expression of an alternative spliced, inactive transcript lacking exons 4 and 5 [32]. In addition the allele A of -31118 G/A SNP has been associated with reduced CD36 expression, lies between two alternative CD36 promoters, 1C and 1A [35]. It has been suggested that the 27645Ins/Del and 30294G/C polymorphisms located in the 3′-UTR could determine decreased mRNA stability [32].

In this research, five polymorphisms in the *CD36* gene in haplotypes combinations were associated with high LDL-C and TC levels. This study showed that a haplotype analysis with five variants in the *CD36* receptor gene may explain the lipid profile variation more than a single variant. Similarly, in the Caucasian population have studied the five polymorphisms in the *CD36* receptor gene and were found in high linkage disequilibrium and a common haplotype at the *CD36* locus was associated with high free fatty acid levels and increased cardiovascular risk [32]. However, further investigations are needed to confirm our findings and demonstrate the mechanisms underlying such associations.

Nonetheless, the results of association studies must always be interpreted with caution, especially when multiple comparisons are performed, and replication in other populations is needed before a link between *CD36* variants, dyslipidemias and cardiovascular disease.

Finally, some limitations of our study should be considered. Although this study had sufficient statistical power to detected large effects resulting from common alleles, the power to evaluate small effects due to rare alleles was limited. However, small genetic effects can be expected because of the complexity of lipid metabolism. Second, we could not completely exclude the influence of factors such as consumption of alcohol and tobacco on lipid levels.

## Conclusions

The study provides evidence of a genetic association of *CD36* haplotypes with the variability in LDL-C and TC levels in a sample of normal-weight subjects. The *CD36* gene may be a candidate susceptibility to dyslipidemia in Mexican population.

## Methods

### Subjects

A total de 232 unrelated normal-weight subjects of 18 to 25 years old, from the state of Guerrero, Mexico. The participants were randomly selected considering their body mass index (BMI) of 18.5 to 24.9 kg/m^2^. There were 157 women and 75 men, none of them had medication with lipid-lowering drugs such as statins or fibrates. The participants signed informed consent forms, and the protocol was approved by the Research Ethics Committee of the University of Guerrero.

### Blood pressure

Blood pressure was measured in the sitting position with the use of an automatic sphygmomanometer on the left arm after 10 min rest. The systolic blood pressure (SBP) and diastolic blood pressure (DBP) were calculated from two readings with a minimal interval of 10 min. Hypertension was defined as mean SBP ≥140 mmHg and/or DBP ≥90 mm Hg [36].

### Biochemical analysis

A venous blood sample of 5 mL was obtained from each subject after at least a 12 hours fasting. All serum lipid levels and glucose were determined by enzymatic methods with commercially available kits (spinreact). Abnormal biochemical levels were identified when total-cholesterol (TC) > 200 mg/dL, TG ≥ 150 mg/dL, LDL-C > 100 mg/dL, HDL-C < 40 mg/dL and glucose >100 mg/dL, based on the criteria of the National Cholesterol Education Program (NCEP) Expert Panel on Detection, Evaluation, and Treatment of High Blood Cholesterol in Adults (Adult Treatment Panel III) [5].

### Determination of serum oxLDL

An enzyme-linked immunosorbant assay (ELISA) for oxLDL (Mercodia Oxidized LDL ELISA) was performed, according to the manufacturer’s instructions using coated microtitration strips of 96-well plates, serum was diluted 1/6561, and incubated at room temperature for 2 h in plates precoated with oxLDL-lgG. After six washings, the plates were incubated with an anti-apolipoprotein B (apoB) monoclonal antibody at room temperature for 30 minutes. After the removal of unbound conjugates by washing the samples six times, tetramethylbenzidine (TMB) was added to the wells as a chromogenic substrate. The mixture was incubated at room temperature in the dark for 15 minutes. Color development was stopped via a stopping solution, and absorbency was measured at 450 nm within 30 minutes. The oxLDL was calculated by constructing a standard curve using the standards included in the kit. The oxLDL concentrations in the samples were quantified in biomedical units as defined by the manufacturer.

### Genotyping

Genomic DNA was extracted from leukocytes in samples of whole blood and was stored at -20°C until analysis. Genotyping of the five polymorphisms -33137A/G (rs1984112), -31118G/A (rs1761667), -22674 T/C (rs2151916), 27645 Ins/Del (rs3840546) and 30294G/C (rs1049673) was performed by polymerase chain reaction and restriction fragment length polymorphism (PCR-RFLP). The five polymorphisms in the *CD36* gene were selected for genotyping based on previously reported association with free fatty acids and triglycerides, whose minor allele frequency >5% and one insertion/deletion polymorphism [32]. Primers for the polymorphims were designed and restriction enzymes (REs) were identified using the Primer 3 and NEB cutter softwares respectively. Unique National Center for Biotechnology Information (NCBI) Build 37 chromosome and base pair locations may be obtained from Ensembl Variation Build 59 (which is based on dbSNP Release 131). Details including the location of polymorphisms in the CD36 gene, primer sequences and REs with product sizes are presented in Table 5.

**Table 5:** **- Characteristics of the polymorphisms studied in the *CD36 *** **gene**

**Polymorphisms**	**Primer sequence**	**Annealing temp. (°C)**	**Product size (bp)**	**Restriction enzyme/allele sizes**
-33137A/G	F: 5′-CATGCAGCTCTGTTTTATGTGAG-3′	60	159	*MseI*
				AA 67, 56, 29, 7
(rs1984112)	R: 5′-CCCCATCTCTTAGGCCCGTGACA-3′			AG 85, 67, 56, 29, 7
				GG 85, 67, 7
*-*31118G/A	F: 5′-CAAAATCACAATCTATTCAAGACCA-3′	58	190	*HhaI*
				AA 190
(rs1761667)	R: 5′-TTTTGGGAGAAATTCTGAAGAG-3′			GA 190, 138, 52
				GG 138, 52
-22674 T/C	F: 5-′TCTTGCTGGGCCCTGCCCA-3′	68	452	*Hpy 188I*
				TT 452
(rs2151916)	R: 5′-TGTTTGCCCCAAGTGCTGGGTC-3′			TC 452, 430, 22
				CC 430, 22
27645Ins/Del16	F: 5′-GGGACCATTGGTGATGAGAAGG-3′	68	563	Ins/Ins
			563, 547	Ins/Del
			547	Del/Del
(rs3840546)	R: 5′–TTGGAAAATGCACGGCCAGCA-3′			
30294G/C	F: 5′-ACGCTTGGCATCTTCAGAATGCT-3′	60	465	*Mnll*
				GG 331, 134
(rs1049673)	R: 5′TGAACCCCTGCTCAAGAAACAGAGT-3′			GC 331, 265, 134, 66
				CC 265, 134, 66

Each reaction system of a total volume of 24 μL, containing 100 ng of genomic DNA, 0.2 mM of each primer, 0.2 mM dNTPs, and 2.0U of *Taq* polymerase (Invitrogen Life Technologies). The PCR products were digested with the respective restriction enzymes, and were visualized on 6% polyacrylamide gels stained with silver nitrate 2%.

### Statistical analysis

The statistical analyses were done with the statistical software package SPSS 15.0 and STATA software 9.0. Quantitative variables were expressed as medians and 25^th^ to 75^th^ percentiles or 5^th^ to 95^th^ percentiles; the significance of differences between groups was determined using Wilcoxon-Mann Whitney or Kruskal Wallis test. Qualitative variables were expressed as percentages. Allele and genotype frequencies were determined by calculating Hardy-Weinberg equilibrium, difference in genotype distribution between the groups female and male was obtained using the chi-square test, a *P* value of less than 0.05 was considered statistically significant. For analysis linkage disequilibrium of polymorphisms in *CD36* gene and the association between haplotypes and lipid phenotypes were analysed using the software program SHEsis (http://analysis.bio-x.cn/myAnalysis.php) [37].

## Competing interests

HBM is Editor-in-Chief of the Journal of Foot and Ankle Research. It is journal policy that editors are removed from the editorial decision making processes for papers they have co-authored. The remaining authors declare that they have no competing interests.

## Authors’ contributions

LERA carried out genetic analysis and writing the manuscript. ABSB performed laboratory measurements and quality control. IPGG performed the statistical analysis. LSG and JFMV participated in the critical revision of the manuscript. IPR conceived the study and participated in manuscript preparation. All authors read and approved the final manuscript.
